# Inferring Animal Densities from Tracking Data Using Markov Chains

**DOI:** 10.1371/journal.pone.0060901

**Published:** 2013-04-22

**Authors:** Hal Whitehead, Ian D. Jonsen

**Affiliations:** Department of Biology, Dalhousie University, Halifax, Canada; Universita' del Piemonte Orientale, Italy

## Abstract

The distributions and relative densities of species are keys to ecology. Large amounts of tracking data are being collected on a wide variety of animal species using several methods, especially electronic tags that record location. These tracking data are effectively used for many purposes, but generally provide biased measures of distribution, because the starts of the tracks are not randomly distributed among the locations used by the animals. We introduce a simple Markov-chain method that produces unbiased measures of relative density from tracking data. The density estimates can be over a geographical grid, and/or relative to environmental measures. The method assumes that the tracked animals are a random subset of the population in respect to how they move through the habitat cells, and that the movements of the animals among the habitat cells form a time-homogenous Markov chain. We illustrate the method using simulated data as well as real data on the movements of sperm whales. The simulations illustrate the bias introduced when the initial tracking locations are not randomly distributed, as well as the lack of bias when the Markov method is used. We believe that this method will be important in giving unbiased estimates of density from the growing corpus of animal tracking data.

## Introduction

The tracking of animals is immensely informative. Animals can be followed visually, acoustically, using their own tracks (e.g. in snow), or, as is most common these days, by means of attached telemetric tags. We may learn of movements, habitat use, foraging patterns, behavior, social structure and life history, as well as how the animals respond to natural and anthropogenic changes to their environment over a range of scales. However, one of the most basic measures which ecologists and managers use and need, relative density, is rarely available from tracking data in an unbiased form. The problem is that the locations of the tracked animals are biased towards where the tracking started—the release location in the case of tagged animals—and there has been no technique available to remove the bias. The distribution of real tracks can be compared with that of randomized tracks [1], but it is not usually clear what constitutes a meaningful “random track”.

These “start-biases” constitute a major drawback, substantially limiting the utility of the large quantities of often hard-won tracking data. Conversely, knowledge of how animals use their habitats is considerably less than what it might be. Management and conservation efforts suffer accordingly.

One way of envisaging an animal track is as a Markov chain, with animals moving from habitat cell to habitat cell. In this vein, Pedersen et al. [2] used a hidden Markov-chain model to estimate residency and behavior from tracking data. Here we introduce a simple Markov-chain method that produces unbiased measures of relative density from tracking data. The density estimates can be over a geographical grid, or relative to environmental measures such as water depth or habitat type, or a combination of these. We explain the theory behind the method, and how it can be used in practice. We illustrate its successful use both using simulated data and real tracks of animals. Finally, we discuss a few cautions in the use of the method, as well as possible extensions.

## Methods

### Ethics statement

The research on sperm whales was conducted under a permit from the Galapagos National Park Service and approved by the Dalhousie University Committee on Laboratory Animals.

### Markov method for inferring density

First we divide the habitat into a finite number of cells. These could be spatial rectangles, or different habitats defined by values of environmental measures, or combinations of these. We then make two assumptions:

1. The tracked animals are a random subset of the population in respect to how they move through the habitat cells.
2. The movements of the animals among the habitat cells form a time-homogenous Markov chain. In other words, the probability that an animal in cell *j* moves into cell *i* at time *t* (*p_ji_*) is independent of *t*, and where the animal was at time *t*-1.

If the probability that an animal randomly chosen from the population is in cell *i* at any time is π*_i_*:
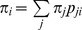



Then, from assumption 2, π = {π*_i_*} satisfies [3]:
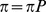
where **P** = {*p_ji_*}. Thus **π** is the left eigenvector of the transition matrix, **P**, associated with eigenvalue 1.0.

As **π** is the unbiased estimate of the proportion of time animals spend in each cell, and thus the proportion of the population in the different cells (from assumptions 1 and 2), this provides a methodology for the estimation of relative density from tracking data.

To implement this method, tracks are divided into steps of equal duration. *n_ji_* is the number of steps that start in cell *j* and end in cell *i*. Steps can start and end in the same cell, so *n_ii_* can be greater than zero. These steps can come from a number of tracks of different, or the same, animals. Then, *p_ji_* can be estimated by:
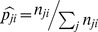



The relative numbers in the different cells come from the left eigenvector of the estimated transition matrix, 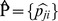, associated with eigenvalue 1.0. Relative densities are then estimated from the corresponding element of this eigenvalue, divided by the area of the cell. These estimated relative densities can be standardized in any way that makes sense, but it may often be appropriate to normalize them so that they sum to one. It may also make sense to have an “exterior” cell for animals leaving the main study area.

### Illustration using simulated data

We simulated the method in a wide range of conditions, but to illustrate its general performance we present four of these simulations. In each of these simulations, 300 agents made uncorrelated random walks [4] over an *x-y* plane with 1,000 moves. The study area was a 1 unit by 1 unit square centered on [0.5, 0.5], although individuals could move outside the study area, or back into it. Two types of study area were simulated:

1. “Homogeneous”, in which all moves are of length 0.05. Here the ideal, expected, distribution is homogeneous across the study area.
2. “Quadrants”, in which move lengths were 0.05/√1.5 in the quadrant with *x*>0.5 and *y*>0.5, 0.05/√0.5 in the quadrant with *x*≤0.5 and *y*≤0.5, and 0.05 in the other two quadrants. These differences in move length, in other words the speed of the agents, give relative densities, ideal distributions, of 0.5, 1.0, 1.0, 1.5 in the four quadrants [4].

We considered two ways in which tracking was initiated:

1. tracks started at randomly-chosen positions within the study area;
2. tracks started at randomly-chosen positions within 0.1 units of the center of the study area ([0.5, 0.5]).

**Figure 1:**
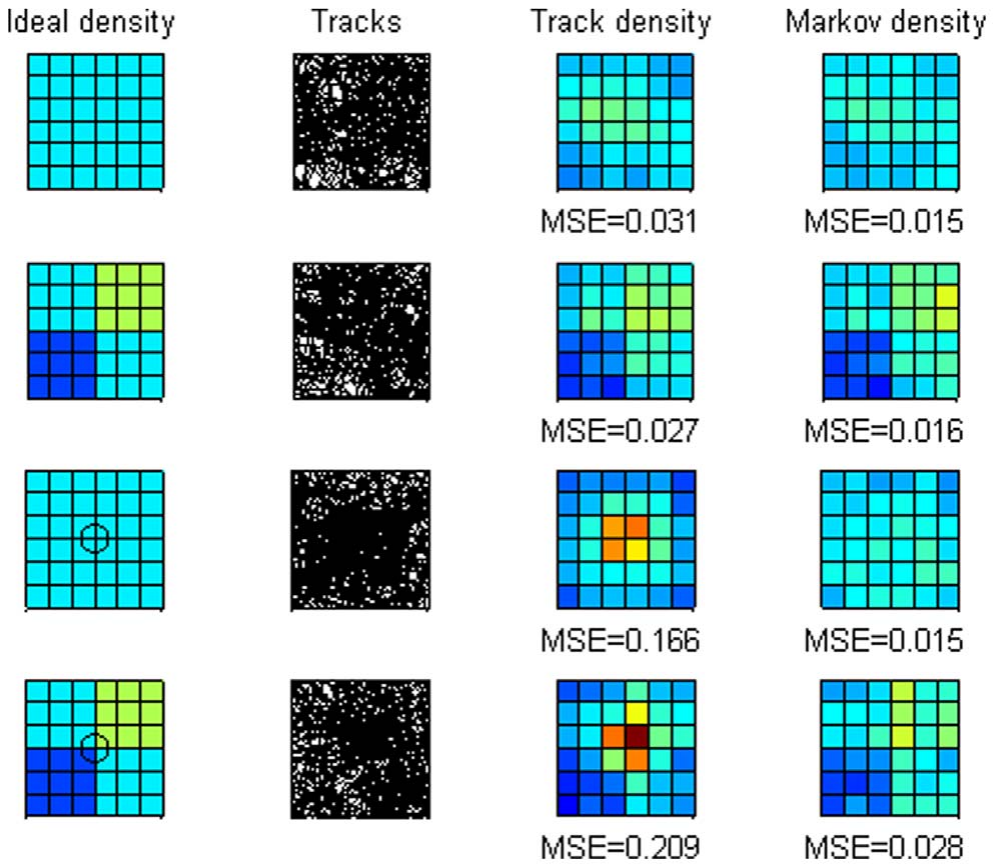
Estimating density from simulated data. Results of four simulations with uniform (rows 1 and 3) or quadrant (rows 2 and 4) density distributions, as well as random (rows 1 and 2) or central (rows 3 and 4) initial tracking positions. Each row indicates the ideal density of agents in the study area, the tracks of 50 of the agents, and the estimated densities from summing track positions (“Track density”) and the Markov technique (“Markov density”). The concordance between the ideal densities and estimated densities is indicated by the mean square error (“MSE”). All density plots use the same normalized color scale ranging from dark blue (near zero) to turquoise (medium) to dark red (maximum).

The track data for each simulation were the positions of the agents after each 20 moves. To estimate relative density, the study area was divided into 36 square cells, plus one “exterior” cell for all regions outside the study area. For each of the four simulations, with the two types of study area, and two tracking start types, we present (Fig. 1): a graphical representation of the ideal, actual density of agents over the study area (“Ideal density”); the tracks (just 50 shown for clarity); estimates of relative density obtained by simply summing the number of track positions in each cell (“Track density”); and estimates of relative density obtained using the Markov eigenvector technique described above (“Markov density”). The estimates of relative density were normalized to have the same mean as the ideal densities, excluding the “exterior” cell. Performance of the two techniques was evaluated from the mean (over cells within the study area) squared error of the estimated relative density in a cell compared with its ideal value.

### Illustration using real data

We also illustrate the method using real tracking data that come from tracks of groups of female and immature sperm whales (*Physeter macrocephalus*) off the Galapagos Islands, Ecuador, between 1985–1995 (study area: 1°S - 1°N; 90° 30′W - 92° 30′W) [5]. The groups of whales were tracked both visually (mainly daylight) and acoustically by listening for the sounds of the whales (mainly at night time) from 10–12 m auxiliary sailing vessels [6]. Positions were determined by SATNAV or GPS (Global Positioning System), and were interpolated every six hours. There were 57 tracks containing 460 locations (i.e. 115 24-hr days tracking).

**Figure 2:**
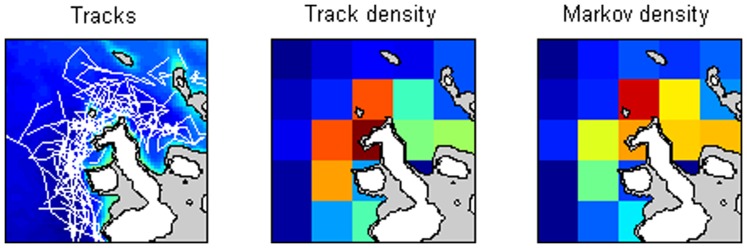
Estimating spatial sperm whale distributions. Distribution of groups of sperm whales off the Galapagos Islands (1°S - 1°N; 90° 30′W - 92° 30′W, so the cells are 44.5 km square) from tracking data (shown at left, with water depths); estimated densities from summing tracking positions (“Track density”) and the Markov technique (“Markov density”). Only waters greater than 1,000 m deep were considered. Islands are shown in white, and waters less than 1,000 m deep in grey. The density plots use the same normalized color scale ranging from dark blue (near zero) to turquoise (medium) to dark red (maximum).

The study area was divided into 25 square cells, plus one exterior cell. Waters less than 1,000 m deep were masked out. We plot, in Fig. 2, the estimated densities in the study area: the track density from the number of tracking locations divided by area within the cell greater than 1,000 meters deep, and the Markov density, also using the usable area in each cell as a divisor. We also analyzed the same data using depth-delineated cells. The depth ranges chosen were 0–750 m, 750–1,250 m, 1,250–1,750 m, and 3,250–3,750 m.

## Results

In the simulations, the Markov density estimates were always closer to the ideal densities(homogeneous across the study area in the “homogeneous” case; or the quadrants having densities proportional to 1, 2, 2, and 3 in the “quadrants” case) than those using the track positions directly (Fig. 1). When the track started in the center of the study area the advantage of the Markov methodology was substantial, with nearly an order of magnitude less mean square error.

**Figure 3:**
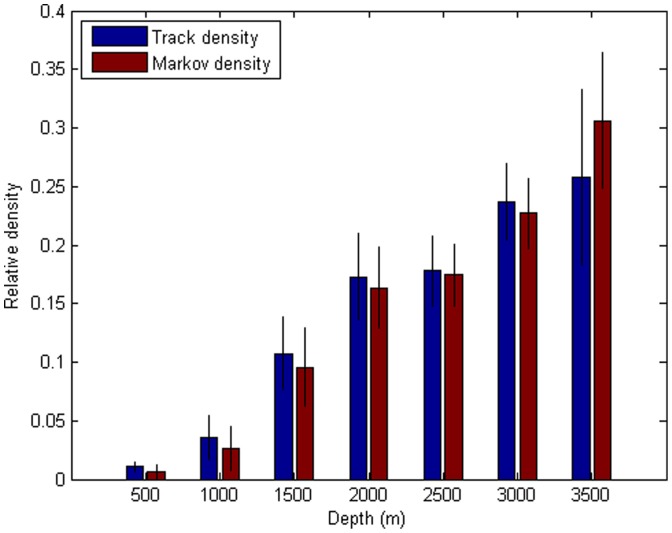
Estimating sperm whale depth distributions. Depth distribution of groups of sperm whales off the Galapagos Islands (1°S - 1°N; 90° 30′W - 92° 30′W) from summing tracking positions (“Track density”) and the Markov technique (“Markov density”). Errors bars show standard errors estimated by the nonparametric bootstrap method (1000 bootstrap replicates of tracks).

For the tracking data of the sperm whales off the Galapagos Islands, the Markov estimates suggest higher density to the north of the principal islands, compared with the track estimates that highlight areas to the west of the islands. For logistic reasons, many of the tracks started to the west of the islands, which explains the bias in the track estimates. The estimates of the proportion of the population using each depth category are shown in Figure 3. The Markov analysis indicates a greater preference for the deepest waters, and avoidance of the shallowest, compared with the simple track estimates. Tracks were preferentially initiated in shallow waters, explaining this bias.

## Discussion

For the Markov method to produce useful estimates of density there need to be reasonable numbers of transitions between the cells. This usually requires considerable tracking data, and might be viewed as a drawback of the method. However, it reflects a general issue: obtaining reasonably accurate estimates of the relative densities of animals over any habitat by sampling needs large sample sizes whatever the method. The Markov method is imprecise with few data, but so will be any other method, and large numbers of tracking locations may only represent a small amount of independent data if the locations have considerable autocorrelation.

An important assumption of the method is that the tracked animals are a random subset of the population in the manner by which they move through the habitat. If they are not, then the Markov method may not produce useful results. For instance if members of the population have individual home ranges within the study area, and tracks are only commenced in one part of the study area, then densities away from the locations where tracks commenced will be considerably underestimated.

We further assume (assumption 2) that movements must form a first-order, time-homogeneous Markov chain. Therefore, if there is second or higher order dependence, the method will be biased. For instance we found that in our simulations, using small step-lengths (considerably less than the size of the cells) removed the advantages of the Markov method, as now there was second-order Markov dependency: agents would tend to move back and forth between adjacent cells if step lengths were small relative to cell size. There are several possible tests for second-order dependence [7]. However, a simple approach that should detect the most likely violations of second-order independence in tracking data is to compare the number of triplets of consecutive locations in which the first cell is the same as the third (e.g. *jij*), 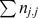, the “returning triplets”, with the expected number:
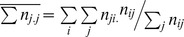



The observed and expected numbers of returning triplets can be compared using a likelihood-ratio G test or chi-squared test. When we followed this procedure with the simulated data, the observed and expected numbers of returning triplets were not significantly different in any of the runs presented in Fig. 1. However when the step size was reduced from 20 moves to 10, so that the step size became considerably less than the cell size, these differences became significant; the agents were preferentially returning to cells from which they had just come. The same test found no significant second-order dependence in the sperm whale data, either spatially (actual returning triplets = 99; expected = 106.2; G =  0.79 with 1 df; P = 0.3739) or with depth (actual returning triplets = 124; expected = 109.4; G =  3.06 with 1 df; P = 0.0801).

We have presented the densities as uniform over the cells in Figs 1–3. However, this will rarely be the case. Densities can be interpolated between cells.

Estimates of any measure have little validity without an estimate of precision. We suggest using bootstrap or jackknife [8] methods to estimate variability in tracking-derived density estimates, bootstrapping or jackknifing on the different tracks. We show nonparametric bootstrap estimates of error in the depth distribution of the sperm whale groups in Fig. 3.

Finally, an unstated assumption is that the tracking data are accurate. Small errors in positions, for instance those caused by interpolating between irregularly-collected locations to obtain steps of uniform duration, will likely have little effect on density estimates especially with large data sets. However, large errors, such as those that occur with light-level geolocation and Argos satellite locations need to be removed [2], [9]. Indeed, when large location errors are known or suspected to exist in the tracking data, we advocate an approach where the Markov method is coupled with state-space filtering of location data [2], [9]. However, variability in the durations of tracks of individual animals should not be an issue with the Markov method.

### Software

In the Supporting Information, we provide MATLAB (File S1.zip) and R (File S2.zip) code for estimating densities using the method described. They take as input: a numerical list of the sequential cells visited during the tracks; a list of the start points of the different tracks; and the areas of the different cells (a final “external cell” without a designated area is an option). The routines can perform the second-order Markov dependency test outlined in the text, as well as make bootstrap estimates of standard errors of density estimates for each cell. They output the estimated Markov density and track position density for each cell.

## Conclusions

Using simulated data and non-invasive visual tracking data of whales, we have shown how a Markov chain approach can reduce bias in relative density estimates from animal tracking data. Our approach easily accommodates more ubiquitous tracking data obtained via electronic tags such as light-based geolocation tags and satellite-based location (Argos and GPS) tags. In recent years, electronic tracking datasets have grown substantially in size, both in number of species and individuals tracked, and attention within marine ecology has turned to using these datasets as alternatives to catch-based or survey-based distribution and relative density information [1], [10]. It is, however, logistically impossible to randomize animal tagging and release locations in large-scale tracking projects and, as a consequence, bias in resulting distribution and relative density estimates is to be expected. We strongly advocate the use of Markov density estimates, which can easily be implemented by ecologists, or similar approaches to reduce this important source of bias.

## Supporting Information

File S1 **MATLAB code for estimating densities using the Markov method.** Includes MATLAB code (markovdens.m), instructions for use (Matlab_readme.txt) and test data set (testset.mat).
(ZIP)

File S2 **R code for estimating densities using the Markov method.** Includes R code (markovdens.R, mdensity.R, secondtest.R), instructions for use (README.txt) and 4 test data sets (radius_quadR.txt, etc.).
(ZIP)
